# Improved oil recovery in nanopores: NanoIOR

**DOI:** 10.1038/srep28128

**Published:** 2016-06-20

**Authors:** James Moraes de Almeida, Caetano Rodrigues Miranda

**Affiliations:** 1 Centro de Ciências Naturais e Humanas, Universidade Federal do ABC, Santo André, SP, Brazil; 2 Instituto de Física, Universidade de São Paulo, São Paulo, SP, Brazil

## Abstract

Fluid flow through minerals pores occurs in underground aquifers, oil and shale gas reservoirs. In this work, we explore water and oil flow through silica nanopores. Our objective is to model the displacement of water and oil through a nanopore to mimic the fluid infiltration on geological nanoporous media and the displacement of oil with and without previous contact with water by water flooding to emulate an improved oil recovery process at nanoscale (NanoIOR). We have observed a barrier-less infiltration of water and oil on the empty (vacuum) simulated 4 nm diameter nanopores. For the water displacement with oil, we have obtained a critical pressure of 600 atm for the oil infiltration, and after the flow was steady, a water layer was still adsorbed to the surface, thus, hindering the direct contact of the oil with the surface. In addition, oil displacement with water was assessed, with and without an adsorbed water layer (AWL). Without the AWL, the pressure needed for oil infiltration was 5000 atm, whereas, with the AWL the infiltration was observed for pressures as low as 10 atm. Hence, the infiltration is greatly affected by the AWL, significantly lowering the critical pressure for oil displacement.

Fluid infiltration and flow in minerals can have important implications to understand underground aquifers, oil and shale gas reservoirs. In those systems, the fluid is within pores with different geometries and scales, varying with the nature of the geological reservoirs and their deepness. It is known that the porosity of the reservoirs decreases as a function of depth, due to the increasing vertical pressures in such environment^1^. This effect can be quite severe, to the point that up to 80% of the porosity is accounted for pores ranging from 0.5 to 100 nm diameter (4500–5600 m depth)^1^. Thus, the nanoporosity can be very important as it interconnects larger pores and control the permeability of such reservoirs^1^.

The confined fluids within these nanopores may have different properties from bulk fluids. E.g., water has a rather unique structuring, due to its intermediate and long-range inter-molecular interactions. Under confinement, this structuring is frustrated by surface effects, that disrupts the hydrogen bond network. As a result, new phenomena can emerge, as new phase transitions^2^,^3^, layering near the interface^4^,^5^, a first layer of “immobile water”^6^.

For the nanoscale, the continuum models for fluids may not work^7^, thus the use of a atomistic description is needed. Molecular dynamics simulations are useful to simulate the fluid flow atomistically, with several examples in the literature. Botan *et al.*^7^ have described the brine flow within clay (montmorillonite) slit pores, ranging from 2 to 9 nm widths. The authors observed that, the region that was perturbed by the surface was up to 1.2 nm long, the densities and velocities in that region varied considerably. The obtained viscosities, were similar to the bulk water viscosity, considering a region of constant density away from the surface. Zhu *et al.*^8^ have simulated water flow between graphene sheets, they have observed that the water flux depends linearly on the pore area, for large enough pores. For the smaller pores (around 17 Å) the flow rate get a non-linear dependency with the pore area, they observe an increased flow for the smaller pores, an important surface effect, as the smaller pores have a greater quantity of water molecules that are influenced by the surface. Xu *et al.*^9^ have explored the effect of wall roughness on the fluid flow in carbon nanotubes, with their molecular dynamics simulations, they have obtained larger shear stress and nominal viscosities for higher roughness, thus the roughness induces a flow resistance in the nanopores, and, the smaller the pore is, the higher is the influence of the roughness.

In our work, we explore the fluid flow on silica nanopores. The simulated nanopores are tailored to mimic real conditions of rock reservoirs with rough surfaces and two distinct passivation and diameters close to 4 nm. We simulate several cases of fluid infiltration: 1) Water and oil infiltration on empty nanopores. 2) Oil infiltration on water filled nanopores. 3) Water infiltration on oil filled nanopores, with and without an adsorbed water layer between the pore surface and the oil. We explore the different pressure differences needed for the fluids to infiltrate, the fluid structuring, interfacial tensions and viscosities inside the nanopores. The final goal of the work is to be able to simulate the oil extraction from a nanopore, namely Nano Improved Oil Recovery (NanoIOR).

## Methodology

We have performed classical molecular dynamics (MD) simulations as implemented in the Large atomic/molecular massively parallel simulator (LAMMPS) package^10^. The chosen inter-atomic potential were, Cruz-Chu^11^ for the amorphous silica matrix, CHARMM^12^ for the oil molecules and SPCE/FH^13^ for the water molecules. In order to combine different atomic species interactions, the Lorentz-Berthelot^14^,^15^ combining rules were employed. All the systems had periodic boundary conditions, in the three directions. The reciprocal space particle-particle particle-mesh (PPPM) method^16^,^17^ was adopted for long-range electrostatic interactions with a cutoff of 10 Å for the van der Waals interactions. A timestep of 0.5 fs was used for all the MD calculations, with velocity-Verlet integration algorithm^18^. Temperature and Pressure were controlled by Nosé-Hoover thermostat^19^,^20^ and Andersen barostat^21^, respectively. The chosen temperature and pressure are 300 K and 200 atm, typical thermodynamical conditions of oil reservoirs. A table with the system sizes and number of atoms is included in the supplementary material (Table S1).

To induce flux on the nanochannels, an external force was applied to the atoms. This force is proportional to each atom’s mass (gravity field). In that sense, all different atomic species will be subject to a constant and equal acceleration. As, there is a thermostat being applied to the system, the external force (in a particular direction), may be nullified while the thermostat tries to reach the thermal equilibrium, by changing the velocities of the atoms. For this reason, the thermostat’s degrees of freedom are reduced, it will be applied only in directions perpendicular to the fluid flow. In addition to that, to avoid the silica matrix being pushed out by the fluid phases, a spring force was applied to the silica atoms, the spring constant used was 1.0 Kcal/(mol.Å^2^).

For the light crude oil or pure water infiltration, the simulation procedure consists in inserting an oil or water slab, 5 nm thick. Then, the MD was run for 2 ns, to let the fluid infiltrate in the nanopore. For the induced flow process, an oil slab was inserted in the system with a water filled pore and an adjacent water reservoir. The systems were then equilibrated for 1 ns, afterwards, the external force was turned on, with the tuned thermostats. Then, the flow took place for at least 5 ns, some cases were run for 10 ns, to make sure the flow was in a steady state, i.e., the radial velocity profile was no longer varying (shown in the Supp. Mat. Fig. S1). After the flow was steady, the external forces were turned off, and the systems where equilibrated for 1 ns, then a production run of 2 ns was used to obtain the desired properties. To simulate the back flow, i.e., water infiltrating back on the nanopore, the simulation is restarted from this point, and, the gravity field is then applied, again, until the flow is steady (5 ns). Finally, another equilibration run (1 ns) is performed, followed by another production run of 2 ns.

In order to characterize the confined fluids (oil and water), several properties were analyzed: the radial density profiles for distinct chemical species, the axial diffusion coefficients and the interfacial tensions. The average radial density profiles (RDPs) were obtained by sampling all the atomic positions inside the nanopores during the production runs (at every 5000 steps). All the RDPs were calculated for a 30 Å radius divided in 50 cylindrical shells, in that way, the silica region will also be included, to have a more clear picture of the rough pore structure. The considered length of the pore for the RDP calculations were from −30 Å to +30 Å of the central point of the pore along the axial direction. Thus, avoiding border and entrance effects.

As the fluids are confined on the radial direction, we have calculated the axial diffusion coefficients (ADC) using the Einstein relation from the Brownian motion, with the adapted dimensionality. It is also interesting to obtain the ADC values of the atoms that are close to the surface, to compare the behavior with the central atoms. Thus, we have divided the atoms in two regions: Region 1, considering atoms within the inner radius (central region) and Region 2, by taking in account the atoms located outside the inner radius (close to the surface). During the whole simulation, some atoms will change region, thus, we have considered only atoms that stayed in a given region for 90% of the simulation time for the mean squared displacement calculation.

We have obtained the pressure tensor for each atom individually according to the method described by Thompson *et al.*^16^. From the obtained pressure tensor, after its transformation to cylindrical coordinates (as described by Kim *et al.*^22^ and Wang *et al.*^23^), we were able to obtain the interfacial tensions with the Irving-Kirkwood method^24^, as described by de Lara *et al.*^25^. The following equation was used to calculate the interfacial tensions:
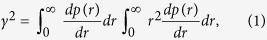
where *γ* is the interfacial tension and *p*(*r*) is the radial component of the pressure tensor. The first integral is evaluated in order to avoid the definition of the limits for each phase of the interface. The pressure tensors are evaluated for all the atoms of the systems, liquid and solid phases. One example of the radial profile of the radial pressure tensor is shown in the supplementary material (Fig. S2).

To obtain both the hydrogen bond number per water molecule and the order parameters S_1_ and S_2_, we have also divided the pore in different cylindrical shells, with a thickness of 0.875 Å. Then, with a snapshot of the dynamics every 5000 steps for at least 2 ns the averages were calculated (minimum 800 snapshots). The chosen hydrogen bond (HB) criteria was geometrical, considering as a HB if the acceptor donor (O…H) distance was lower than 2.5 Å and the OHO angle higher than 100°. The order parameter S_1,2_ is given by the following equation:
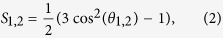
where *θ*_1_ is the angle between the water molecule’s dipole and the normal vector to the surface, and *θ*_2_ is the angle between the water molecule dipole and the pore’s axis. The lower and higher limits of S_1,2_ are: S_1_ = 1 (S_2_ = 1) then *θ*_1_ = 0° (*θ*_2_ = 0°), this means that the water molecule dipole is aligned to the cylinder normal (cylinder axis), and, S_1_ = −0.5 (S_2_ = −0.5) then *θ*_1_ = 90° (*θ*_2_ = 90°) the water molecule dipole is perpendicular to the cylinder normal (axis).

In order to obtain the viscosity of the studied fluids, we have employed the method described by Botan *et al.*^7^. In this method, after a fluid flowing through a pore achieves steady-state flow its velocity profile would reach a parabolic shape. As our systems have cylindrical pores, they will show a radial velocity profile (RVP). With the calculated RVP 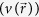, the applied force to induce flux (*F*_*g*_(*z*)) and the fluid density (*ρ*), the viscosity (*η*) can be determined with the following equation:
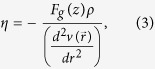
where 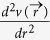 is the second derivative of the RVP. Although, for this expression to work, one has to consider both the viscosity and the density as constants, thus, it should be evaluated far enough from the surface to neglect the variations due to surface effects. To obtain the velocity profile, we have calculated the average velocity as a function of the radius of the molecules that flow through the nanopore. The sampling was for each 5000 steps for at least 3 ns after steady flow (minimum 1200 snapshots), the bin size for the radial discretization was 1 Å.

**Figure 1:**
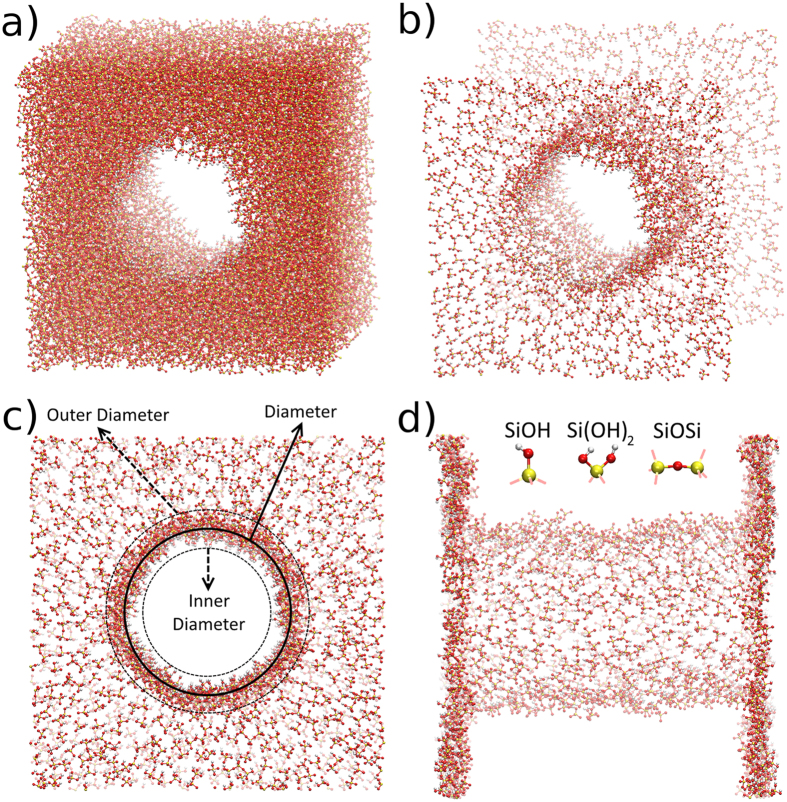
The SiOH-Rich case for the coordination defect free cubic amorphous silica matrix. On (**a**) the full matrix is shown, where yellow spheres are silicon, red are oxygen and white are hydrogens. On (**b**–**d**) only the surface atoms are left, for better visualization of the pore geometry. Three different angles are shown, for a full visualization of the carved silica matrix.

The silica nanopores were carved on a coordination defect free cubic amorphous silica matrix of 9.6 nm side. Since, the initial silica matrix is under periodic boundary conditions, and, the aim of this work is to simulate nanopores with fluid reservoirs along side, the edges of the supercell perpendicular to nanopore’s axis were also carved, leaving two exposed surfaces, as shown in Fig. 1. In order to obtain charge neutral surfaces, the carving process left only oxygen atoms at the surfaces (with no under coordinated Si). One could then, simply add hydrogen atoms to passivate the exposed oxygen atoms, however, amorphous silica surfaces contains SiOH, Si(OH)_2_ and SiOSi (siloxane) terminations. In order to obtain realistic surfaces, a Monte Carlo scheme, as proposed by Makimura *et al.*^26^, was used to passivate the surface. We have explored two different passivations as shown in Table 1.

One can see that the two different passivations have sightly different diameters, this happens because during the carving/passivation process a rough surface is obtained, with inner and outer diameters, as shown on Fig. 1(c). The difference between the inner and outer diameter of the nanopores is about 10 Å, and the diameters showed on Table 1 are the middle point between the inner and outer diameters, the “diameter” shown on Fig. 1(c). It is important to observe that the different passivations leads to different hydrophilicities. It is known that siloxane reconstructed silica surfaces are hydrophobic^27^, whereas terminations on silanol and geminal groups are hydrophilic^28^. As we have all these terminations, with different concentrations, our surfaces will have different hydrophilicities. The SiOH-Rich case is more hydrophilic than the SiOSi-Rich case, because it have a lower concentration of siloxanes in the surface. This can significantly change the fluid interactions with the surface, as will be explored in this work.

The simulations were performed with pure water and light crude oil. The light crude oil was a combination of alkanes, cycloalkanes, and aromatic hydrocarbons molecules with the composition of: 11.14% of hexane, 14.03% of heptane, 16.00% of octane, 20.71% of nonane, 7.25% of cyclohexane, 13.75% of cycloheptane, 4.21% of benzene and 12.91% of toluene, already used in previous works^29^,^30^.

## Results

### Oil and Water Infiltrating Empty Nanopore

**Figure 2:**
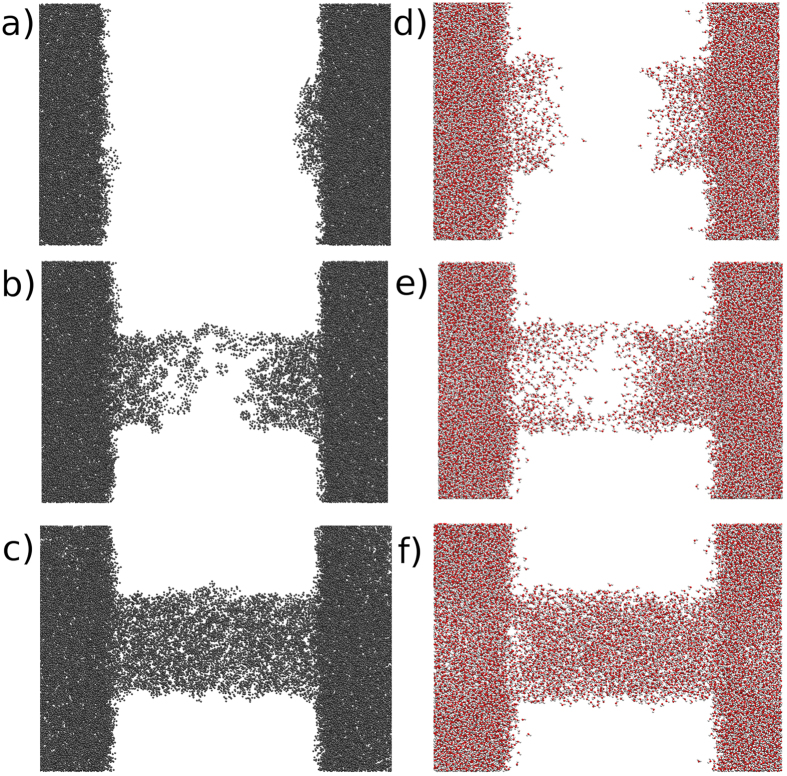
Oil and Water molecules penetrating the SiOH-Rich case nanopore. The silica matrix is removed for better visualization of the fluid infiltration. (**a**) oil at 1 ps, (**b**) oil at 5 ps, (**c**) oil at 0.5 ns, (**d**) water at 1 ps, (**e**) water at 0.1 ns and (**f**) water at 1 ns. Gray molecules are oil, red molecules are water.

Initially, we have explored the fluid infiltration of water and oil on empty (vacuum) silica nanopores. On Fig. 2(d–f), three snapshots of the water infiltration simulation are shown. The water molecules infiltrate quickly, in less than 1 ns, and symmetrically from both sides of the structure. First, the molecules adhere to the surface of the nanopore, and start to occupy “deeper” positions as the simulation goes on. After that, the inner part of the nanopore begin to be occupied, as the water molecules start to structure through hydrogen bonds with those that are already on the surface and with the ones on the reservoir. After 1 ns, the entire nanochannel is filled. The process is spontaneous, and there is no need to apply any forces to induce the infiltration.

On Fig. 2(a–c), the oil infiltration process is shown. Similar to water, the infiltration begins with molecules adhering to the surface, then, the core of the nanopore starts to be filled. However, the oil infiltration is quicker than water, after a half of nanosecond, the pore is completely filled. The water infiltration process is slower because of the hydrogen bond network, that hinder the movement of the molecules. F or the oil, we do not have such interactions, therefore, molecules can infiltrate quicker. For both the water and oil cases, the passivation does not change the infiltration process, since the observed infiltration is very similar.

**Figure 3:**
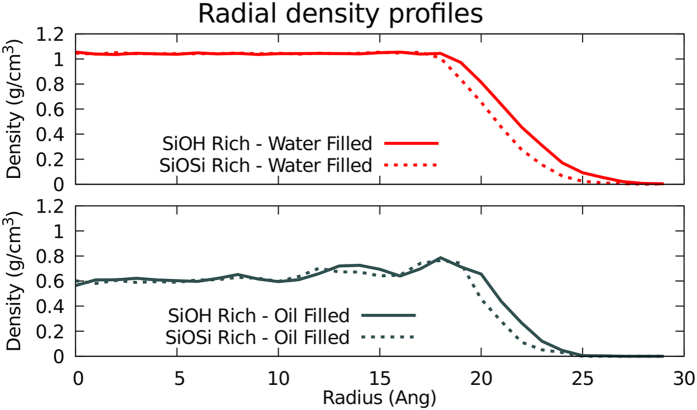
Radial density profile of fluid filled silica nanopores, for both passivations.

**Figure 4:**
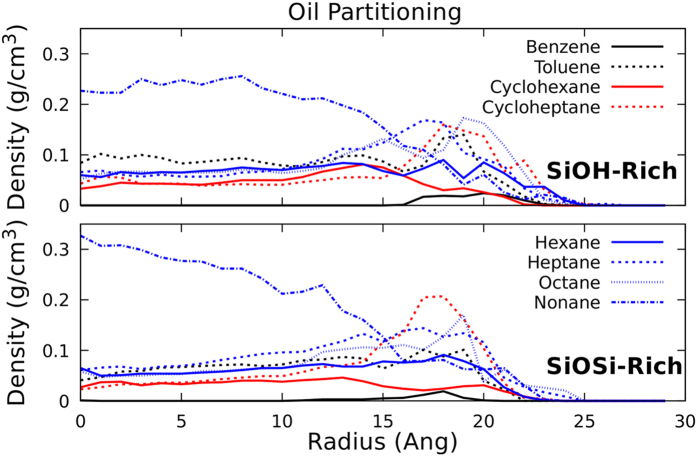
Radial density profile of oil filled silica nanopores. All the different hydrocarbons that compose the oil model are shown.

The radial density profiles (RDP) of the water and oil filled pores were analyzed for both passivations. On Fig. 3, one can see that the systems have very similar molecular distributions for the two studied passivations. The SiOH-Rich case extends to higher radius, as the pore diameter in these cases is 3 Å higher (radius 1.5 Å bigger). The oil model that we are using has several different hydrocarbon molecules, thus, analyzing each species separately can bring new information about the system. The RDP for each hydrocarbon molecule is plotted at Fig. 4, there is a clear accumulation of nonane near the center of the nanopore. In addition, the benzene molecules only appear near the surface. Thus, the lightest oil component tends to be near the surface, and the heaviest stays close to the center of the nanopore. Comparing the two studied passivations, again there are no major differences. In the reservoir, the water and oil densities were 1.03 and 0.65 g/cm^3^, respectively.

For the axial diffusion coefficients (Table 2), for both fluids in region 1 the SiOSi-rich case has lower diffusion coefficients than the SiOH-rich case. Whereas, for the region 2 the diffusion coefficients are higher, again, for both fluids, due to the weaker interaction with the less hydrophilic surface. Also, for both passivations the diffusion coefficient is one to two orders of magnitude lower near the surface (region 2) than in the central region 1, as the molecules have their movement hindered by the interaction with the surface and also by the surface roughness. The self diffusion coefficients for water and oil in the reservoir were 2.71 × 10^−5^ cm^2^/s and 1.95 × 10^−5^ cm^2^/s, respectively.

Whereas, for the calculated interfacial tensions (Table 3), there is a clear difference between the two passivations. As one can notice, the silica/oil interfacial tensions decreases for the SiOSi-Rich case, which is less hydrophilic, whereas, for the silica/water the interfacial tensions are not significantly affected. Thus, the oil is more influenced by the difference in the surface passivations.

### Oil Infiltrating Water Filled Nanopore

**Figure 5:**
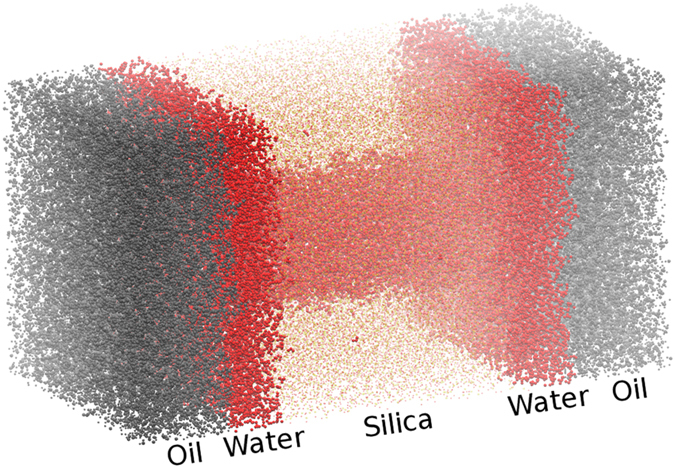
Water filled silica nanopore, with an adjacent water reservoir and also an oil slab on alongside the water reservoir. Red atoms are water, yellow atoms are silica and gray atoms are oil.

In this subsection, we will explore both passivations with an oil/water interface in the reservoir, as shown on Fig. 5. The reservoir has water alongside the silica matrix, and after about 2 nm (for both sides), the oil slab begins, having a thickness of 5 nm. To induce the fluid flux, as explained in the methodology section, a gravity field was applied, only to the oil molecules. The objective is to emulate a pressure difference on the system. The equivalent pressure is obtained by summing up the forces that each atom is subject to, and dividing by the simulation cell area perpendicular to the flow direction. It is also important to consider the geological oil infiltration processes on the rock nanopores, where the pore is initially filled by water or brine, which is displaced by the invading oil. In this way, the correct structuring of the oil, water and rock surface inside the nanopore can be captured and the oil displacement could be simulated at detailed and precise fashion.

**Figure 6:**
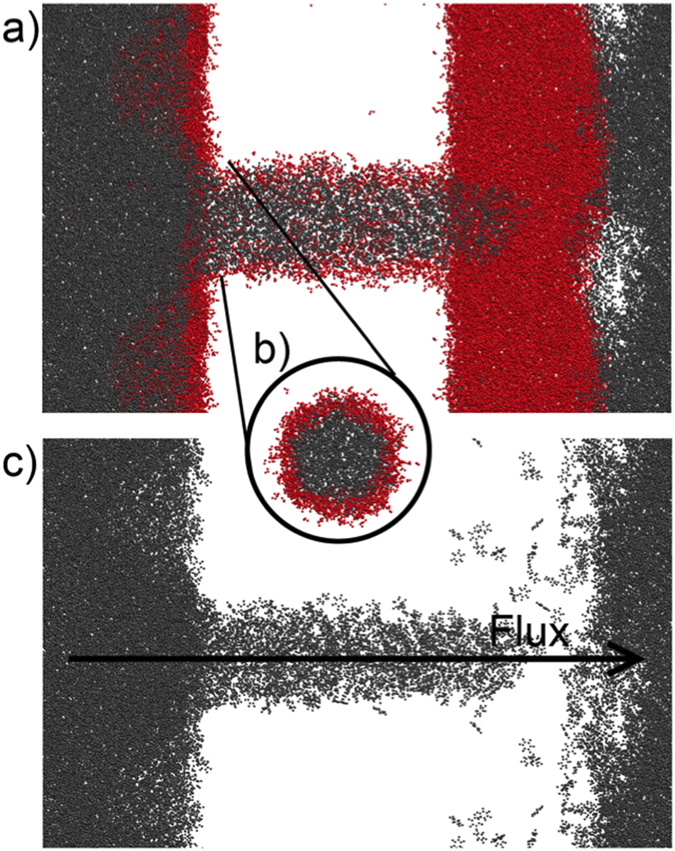
SiOH-Rich Water filled silica nanopore with induced oil flow. On (**a**), the nanopore is shown without the silica matrix for better visualization. On (**b**), only the nanopore region is shown, rotated by 90 degrees to show the remaining water layer. On (**c**), only the oil molecules are shown, to illustrate the infiltration. Red molecules are water, gray molecules are oil.

Different pressures were applied, in order to obtain the critical pressure P_*c*_ for the oil infiltration. No infiltration was observed for both SiOH-Rich and SiOSi-Rich cases when pressures of 200 to 550 atm were applied. For pressures above 600 atm the infiltration was observed for both passivations. After the flow was established, one could see that a water layer remained adsorbed at the pore’s surface, as shown in Fig. 6. It is also important to note that, for 1000 and 1500 atm pressures a dripping effect at the exit end of the nanochannel is observed. Whereas, at 2500 atm pressure, a steady flux with no dripping occurs.

**Figure 7:**
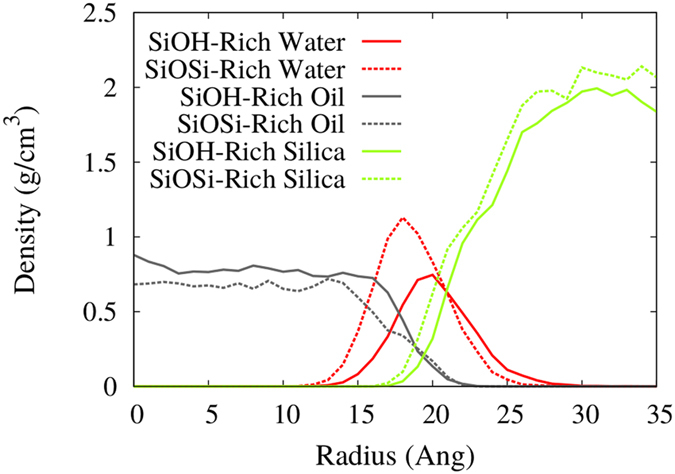
Density profiles of the fluids confined at the nanopores, after flow. SiOH-Rich and SiOSi-Rich cases are shown.

After the flux, the systems were equilibrated for 1 ns, by turning off the external forces, followed by a 2 ns production run. The radial density profiles for both passivations are shown on Fig. 7. The two different passivations have different positions for the peak water densities, again because they have different diameters. The Gaussian-like distribution of the adsorbed water extends to the end of the rough part of the silica (increasing density region of the silica RDP), its width can be related to the characteristic length that the water molecules have reduced mobility^31^,^32^. However, some water molecules can be found at the pore region (silica RDP = 0), hydrogen-bonded to the water within the surface’s indentations. Interestingly the SiOSi-Rich case have a higher density, reaching 1.13 g/cm^3^, contrasting to the 0.75 g/cm^3^ of the SiOH-Rich case. The nanopore radius difference between SiOH-Rich and SiOSi-Rich is 1.5 Å, whereas the peak water density radius differs by 5 Å.

**Figure 8:**
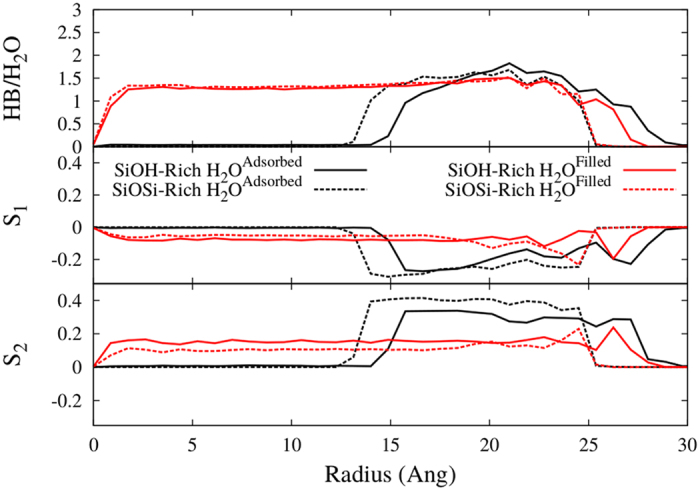
Analysis of the water structuring and ordering, comparing the water filled pores and the oil filled pores with a water layer adsorbed. The first panel shows the number of hydrogen bonds per water molecule, the second panel shows the order parameter S_1_ and the third panel shows the order parameter S_2_, as functions of the radius.

The higher density near the less hydrophilic surface is counter-intuitive and should be looked more closely. To clarify this behavior we have calculated the number of hydrogen bonds per water molecule as a function of the radius, shown in Fig. 8. The SiOSi-Rich case has a very similar shape near the surface for both adsorbed water (SiOSi-Rich H_2_O^*Adsorbed*^) and the water filled reservoir (SiOSi-Rich H_2_O^*Filled*^). Although, the SiOH-Rich H_2_O^*Adsorbed*^ case has a higher number of hydrogen bonds (HBs) per molecule than the SiOH-Rich H_2_O^*Filled*^, and extends for a longer radius. This means that when the water is adsorbed on the more hydrophilic surface, and do not have the inner water layers, that were washed by the oil flux, the HBs with the surface are enhanced, hindering the adsorption of inner water layers, that are more easily removed by the oil flux. The SiOSi-Rich H_2_O^*Adsorbed*^ water do not interact so strongly with the less hydrophilic surface, thus being more accessible to hydrogen bonds with inner water layers, thus the water density keeps increasing for lower radius, as the water-water interaction is stronger. Then, the inner and higher peak is observed.

**Figure 9:**
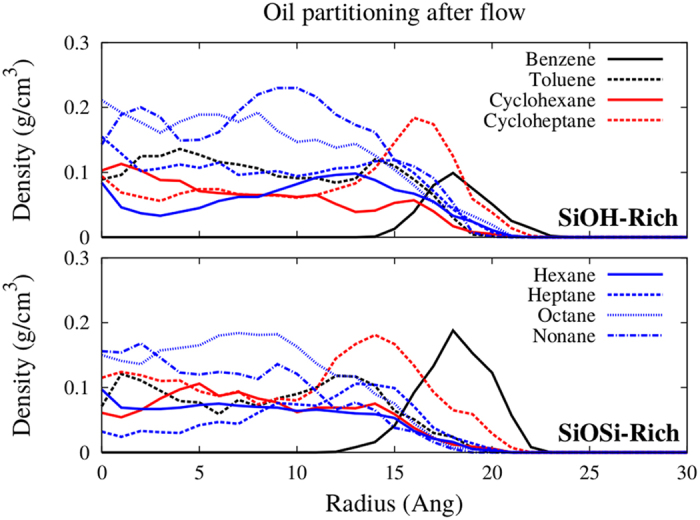
Density profiles of the oil components confined at the nanopores, after flow. SiOH-Rich and SiOSi-Rich cases are shown.

The oil partitioning was also observed for this case, shown on Fig. 9. With the adsorbed water layer, the molecule distribution of the oil changes significantly, comparing with the oil only filled pore (Fig. 4). The different hydrocarbons are more evenly distributed, the nonane does not have a twofold difference in the density as it was the case for the oil only filled pore. Also, there is a higher content of benzene in the oil, and it is highly accumulated at the pore’s surface, with the SiOSi-Rich case having a higher peak near the surface, also cycloheptane accumulation in both cases is more pronounced. Additionally, the interfacial tension were calculated, as shown in Table 3. To the more hydrophilic SiOH-Rich case the interfacial tension increases by 30% with the adsorbed water layer, when comparing to the oil only filled nanopore. Whereas, the less hydrophilic SiOSi-Rich case is not affected by the adsorbed water layer. Thus, the presence of the water layer can change the interfacial tension, thus to calculate it properly one should consider this water layer, because as shown in this subsection, when the oil infiltrates the nanopore a water layer will remain adsorbed, thus, that is likely to be found in oil reservoirs.

The diffusion coefficients were obtained for the equilibrated systems after the flow, as shown in Table 2. The SiOH-Rich case has no water in the central region 1, and in region 2 its diffusion coefficient is one order of magnitude higher than in the region 2 of the pure water filled nanopore. Thus, the adsorbed water is more mobile than the water that is close to the surface in a water-only filled nanopore. That is due to the fact that those water molecules are not involved in a hydrogen bond network with neighboring water, as the the adjacent fluid is oil. Whereas, for the SiOSi-Rich case the diffusion coefficients are similar for both simulations, as the interaction with the surface is already weak. For the SiOH-Rich passivation, the oil in the central region 1 has a diffusion coefficient that is one order of magnitude lower than the pure oil nanopore, whereas, for the SiOSi-Rich passivation the diffusion coefficient is 1.5 times higher than in the pure oil nanopore with the same passivation.

Another point of analysis was the ordering of the confined water molecules. On Fig. 8, we have plotted the order parameters S_1_ and S_2_ as a function of the nanopore radius. The adsorbed water (SiOH-Rich H_2_O^*Adsorbed*^ and SiOSi-Rich H_2_O^*Adsorbed*^) have higher ordering then the water only filled pores (SiOH-Rich H_2_O^*Filled*^ and SiOSi-Rich H_2_O^*Filled*^). After the surface disturbance the values for both passivations approach −0.3 (*θ* = ±68.6°), and that is close to the dipole-OH bond angle of 54.7°, thus, the hydrogens tend to be pointing to the cylinder surface. In addition, the SiOH-Rich case is more perturbed by the surface, due to the higher hydrophilicity. Closer to the surface the angles are lower (oscillate around ±61° from r = 18 to 23 Å), then the molecules are oriented even closer to the dipole-OH bond angle. The S_1_ for the SiOH-Rich H_2_O^*Adsorbed*^ case has contributions for higher radius then the SiOH-Rich H_2_O^*Filled*^ case, reflecting the hydrogen bond behavior, the last peak also have a higher moduli for the adsorbed water, contrasting with the SiOSi-Rich case, were both SiOSi-Rich H_2_O^*Adsorbed*^ and SiOSi-Rich H_2_O^*Filled*^ have the same peak height. Hence, it corroborates to the higher influence of the surface for the SiOH-Rich H_2_O^*Adsorbed*^ case. The SiOSi-Rich H_2_O^*Adsorbed*^ case difference in relation to the SiOSi-Rich H_2_O^*Filled*^ case are the higher values of S_1_ away from the surface, meaning that the water is more oriented due to the lack of inner water molecules to form hydrogen bonds, the same stands for SiOH-Rich H_2_O^*Adsorbed*^ and SiOH-Rich H_2_O^*Filled*^. The S_2_ for the only water filled nanopores (SiOH-Rich H_2_O^*Filled*^ and SiOSi-Rich H_2_O^*Filled*^) are have an increase near the surface, contrasting with the adsorbed water cases. Therefore, when the pore is full of water, near the surface the water molecules are a bit more tilted in relation to the pore’s axis then in the adsorbed water case. The same is true for S_1_ just for the SiOSi-Rich cases, again showing a lower influence of the surface in these cases.

We have also calculated the viscosity of water and oil flowing through the nanopore, as shown in Table 4. All the viscosities were obtained with the applied pressure of 2500 atm. The obtained velocity profiles were after infiltration equilibration when a steady flow regime is obtained. The water viscosity was calculated when the water was injected in an oil filled pore (with the water layer adsorbed on the surface). To obtain the oil viscosity it was injected in a water filled pore (water only). The obtained viscosities are significantly higher than the values calculated for bulk (using Green-Kubo method^33^), the confinement clearly has an effect on the fluid transport, for both water and oil. Also, the surface passivation has influence on the viscosities, as one can observe that for both water and oil the viscosities are higher for the SiOH-Rich case, thus, the hydrophilicity again plays an important role on the fluid behavior, leading to increased viscosities.

### Water Infiltrating Oil Filled Nanopore

We have simulated two cases of water infiltration in oil filled nanopores, the first was with the adsorbed water layer, obtained from the oil infiltration simulations. The second case was an oil only filled pore, thus we can access the differences in the obtained properties. Again, different pressures were applied to obtain the flow through the nanopore. When there is a adsorbed water layer (AWL), the water infiltrates with applied pressures as low as 10 atm (lowest simulated pressure). Although, when there is no AWL (oil only filled pore) the water does not infiltrate that easily, with applied pressures ranging from 2500 to 4500 atm no water infiltration is observed, only when a pressure of 5000 atm is applied the water is able to displace the oil and infiltrate in the nanopore. In such way, the presence of the AWL is crucial to correctly model the oil infiltration on pores, with a striking difference if it is not taken into account.

## Conclusions

We have simulated nanopores carved on a amorphous silica matrix, with two different hydrophilicities, the SiOH-Rich (higher hydrophilicity) and SiOSi-Rich (lower hydrophilicity). The water and oil infiltration on these nanopores was explored. First, empty (vacuum) nanopores with water or oil adjacent reservoirs were simulated, both water and oil infiltrated quickly (less than 1 ns) on the nanopores. The radial density profiles and oil partitioning were not significantly affected by the different hydrophilicities, neither the water/silica interfacial tension. Although the oil/silica interfacial tension was reduced by 35% for the less hydrophilic case (SiOSi-Rich). After that a reservoir with an oil/water interface was simulated, in order to inject the oil in the water filled nanopores. Initially, we have obtained the critical pressure (P_*c*_) necessary for the oil to infiltrate in the nanopore. For both passivations we have found a P_*c*_ of 600 atm. After the oil infiltration a water layer was still adsorbed to the surface, with a higher density for the less hydrophilic case. The presence of the adsorbed water layer increased the interfacial tension by 30% for the SiOH-Rich case, but the SiOSi-Rich case was not affected. As the main interest of the oil industry is to displace oil through rock pores, we have simulated the water infiltration on oil filled silica nanopores, with and without the adsorbed water layer. The critical pressure for the water infiltration was assessed for both cases, and with the adsorbed layer the infiltration was observed for pressures as low as 10 atm (lowest simulated pressure), whereas, for the oil only filled pore the critical pressure needed was 5000 atm. Remarkably a thin water layer can be critical for the correct description of water infiltration on oil filled pores. This can be crucial for modeling of geological processes at nanopore scale, that can be applied to the oil industry.

## Additional Information

**How to cite this article**: de Almeida, J. M. and Miranda, C. R. Improved oil recovery in nanopores: NanoIOR. *Sci. Rep.* **6**, 28128; doi: 10.1038/srep28128 (2016).

## Supplementary Material

Supplementary Information

## Figures and Tables

**Table 1:** The two different simulated passivations.

**Label**	**Diameter (Å)**	**SiOH terminations**	**Si(OH)**_**2**_ **terminations**	**SiOSi terminations**
SiOH-Rich	39.42	6.65%	44.50%	48.86%
SiOSi-Rich	36.45	30.76%	4.60%	65.71%

SiOH-Rich as a more hydrophilic character, whereas the SiOSi-Rich is less hydrophilic.

**Table 2:** For both passivations the diffusion coefficients for all the simulated cases are shown: Pure water, pure oil, oil with adsorbed water layer on the surface.

**Passivation**	**Region**	**Fluid**	**Fluid in the Nanopore**		
			**Pure oil**	**Pure water**	**Adsorbed water and oil**
SiOH-Rich	1	Water	–	2.03 × 10^−5^	–
SiOH-Rich	2	Water	–	3.10 × 10^−7^	2.63 × 10^−6^
SiOH-Rich	1	Oil	1.35 × 10^−5^	–	1.94 × 10^−6^
SiOH-Rich	2	Oil	1.28 × 10^−7^	–	–
SiOSi-Rich	1	Water	–	1.79 × 10^−5^	–
SiOSi-Rich	2	Water	–	7.70 × 10^−7^	4.01 × 10^−7^
SiOSi-Rich	1	Oil	7.39 × 10^−6^	–	1.11 × 10^−5^
SiOSi-Rich	2	Oil	1.43 × 10^−7^	–	–

All the values are diffusion coefficients with cm^2^/s units. The region 1 is from the center of the nanopore up to the inner radius. The region 2 is for radius bigger than the inner radius.

**Table 3:** Calculated interfacial tension for the different passivations and confined fluids.

**Passivation**	**Interfacial Tensions**		
SiOH-Rich	0.57 ± 0.02 N/m	0.58 ± 0.02 N/m	0.74 ± 0.02 N/m
SiOSi-Rich	0.33 ± 0.01 N/m	0.62 ± 0.01 N/m	0.38 ± 0.01 N/m

**Table 4:** Calculated viscosities for Oil and Water with at 2500 atm for all cases.

**Passivation**	**Water**	**Oil**
SiOH-Rich	2.21 ± 0.03 cP	8.86 ± 0.88 cP
SiOSi-Rich	1.63 ± 0.03 cP	3.12 ± 0.24 cP
Bulk	0.63 ± 0.01 cP	0.41 ± 0.03 cP
Experimental	0.888 cP^34^	–

## References

[b1] Katsube T. & Williamson M. Effects of diagenesis on shale nano-pore structure and implications for sealing capacity. *Clay Miner*. 29, 451–472 (1994).

[b2] Hansen E. W., Stocker M. & Schmidt R. Low-temperature phase transition of water confined in mesopores probed by nmr. influence on pore size distribution. *J*. *Phys*. *Chem*. 100, 2195–2200 (1996).

[b3] Liu L., Chen S.-H., Faraone A., Yen C.-W. & Mou C.-Y. Pressure dependence of fragile-to-strong transition and a possible second critical point in supercooled confined water. *Phys*. *Rev*. *Lett*. 95, 117802 (2005). 16197049 10.1103/PhysRevLett.95.117802

[b4] Bonnaud P. A., Coasne B. & Pellenq R. J.-M. Molecular simulation of water confined in nanoporous silica. *J*. *Phys*.*: Condens*. *Matter* 22, 284110 (2010). 21399282 10.1088/0953-8984/22/28/284110

[b5] Du Z. & de Leeuw N. H. Molecular dynamics simulations of hydration, dissolution and nucleation processes at the *α*-quartz (0001) surface in liquid water. *Dalton Trans*. 22, 2623–2634 (2006). 16804574 10.1039/b516258k

[b6] Bourg I. C. & Steefel C. I. Molecular dynamics simulations of water structure and diffusion in silica nanopores. *J*. *Phys*. *Chem*. *C* 116, 11556–11564 (2012).

[b7] Botan A., Rotenberg B., Marry V., Turq P. & Noetinger B. Hydrodynamics in clay nanopores. *J*. *Phys*. *Chem*. *C* 115, 16109–16115 (2011).

[b8] Zhu C., Li H. & Meng S. Transport behavior of water molecules through two-dimensional nanopores. *J*. *Chem*. *Phys*. 141, 18C528 (2014). 10.1063/1.4898075 25399193

[b9] Xu B., Li Y., Park T. & Chen X. Effect of wall roughness on fluid transport resistance in nanopores. *J*. *Chem*. *Phys*. 135, 144703 (2011). 22010727 10.1063/1.3651158

[b10] Plimpton S. Fast parallel algorithms for short-range molecular dynamics. *J*. *Comput*. *Phys*. 117, 1–19 (1995).

[b11] Cruz-Chu E. R., Aksimentiev A. & Schulten K. Water/silica force field for simulating nanodevices. *J*. *Phys*. *Chem*. *B* 110, 21497–21508 (2006). 17064100 10.1021/jp063896o PMC2517990

[b12] Brooks B. R. *et al.* Charmm: The biomolecular simulation program. *J*. *Comput*. *Chem*. 30, 1545–1614 (2009). 19444816 10.1002/jcc.21287 PMC2810661

[b13] Alejandre J., Chapela G. A., Bresme F. & Hansen J.-P. The short range anion-h interaction is the driving force for crystal formation of ions in water. *J*. *Chem*. *Phys*. 130, 174505 (2009). 19425788 10.1063/1.3124184

[b14] Lorentz H. A. Ueber die anwendung des satzes vom virial in der kinetischen theorie der gase. *Ann*. *Phys*. 248, 127–136 (1881).

[b15] Berthelot D. Sur le mélange des gaz. *C*. *R*. *Hebd*. *Seances Acad*. *Sci*. 126, 1703–1855 (1898).

[b16] Thompson A. P., Plimpton S. J. & Mattson W. General formulation of pressure and stress tensor for arbitrary many-body interaction potentials under periodic boundary conditions. *J*. *Chem*. *Phys*. 131, 154107 (2009). 20568847 10.1063/1.3245303

[b17] Hockney R. W. J. E. Computer Simulation using Particles (Adam Hilger, New York, 1989).

[b18] Allen M. P. & Tildesley D. J. Computer Simulation of Liquids (Oxford University Press, 1987).

[b19] Nosé S. A unified formulation of the constant temperature molecular dynamics methods. *J*. *Chem*. *Phys*. 81, 511–519 (1984).

[b20] Hoover W. G. Canonical dynamics: Equilibrium phase-space distributions. *Phys*. *Rev*. *A* 31, 1695–1697 (1985). 10.1103/physreva.31.1695 9895674

[b21] Andersen H. C. Molecular dynamics simulations at constant pressure and/or temperature. *J*. *Chem*. *Phys*. 72, 2384–2393 (1980).

[b22] Geun Kim B., Sik Lee J., Han M. & Park S. A molecular dynamics study on stability and thermophysical properties of nanoscale liquid threads. *Nanoscale Microscale Thermophys*. *Eng*. 10, 283–304 (2006).

[b23] Wang X. & Zhu R. A method to calculate the surface tension of a cylindrical droplet. *Eur*. *J*. *Phys*. 31, 79–87 (2010).

[b24] Irving J. H. & Kirkwood J. G. The statistical mechanical theory of transport processes. iv. the equations of hydrodynamics. *J*. *Chem*. *Phys*. 18, 817–829 (1950).

[b25] de Lara L. S., Michelon M. F., Metin C. O., Nguyen Q. P. & Miranda C. R. Interface tension of silica hydroxylated nanoparticle with brine: A combined experimental and molecular dynamics study. *J*. *Chem*. *Phys*. 136, 164702 (2012). 22559499 10.1063/1.4705525

[b26] Makimura D. *et al.* Combined modeling and experimental studies of hydroxylated silica nanoparticles. *J*. *Mater*. *Sci*. 45, 5084–5088 (2010).

[b27] Laskowski J. & Kitchener J. The hydrophilic—hydrophobic transition on silica. *J*. *Colloid Interface Sci*. 29, 670–679 (1969).

[b28] Zhuravlev L. The surface chemistry of amorphous silica. zhuravlev model. *Colloids Surf*., *A* 173, 1–38 (2000).

[b29] de Lara L. S., Michelon M. F. & Miranda C. R. Molecular dynamics studies of fluid/oil interfaces for improved oil recovery processes. *J*. *Phys*. *Chem*. *B* 116, 14667–14676 (2012). 23163479 10.1021/jp310172j

[b30] Kunieda M. *et al.* Self-accumulation of aromatics at the oil/water interface through weak hydrogen bonding. *J*. *Am*. *Chem*. *Soc*. 132, 18281–18286 (2010). 21141860 10.1021/ja107519d

[b31] Chiavazzo E., Fasano M., Asinari P. & Decuzzi P. Scaling behaviour for the water transport in nanoconfined geometries. *Nat*. *Commun*. 5, 4565 (2014). 24699509 10.1038/ncomms4565 PMC3988813

[b32] Fasano M., Chiavazzo E. & Asinari P. Water transport control in carbon nanotube arrays. Nanoscale Research Letters 9, 1–8 (2014). 25313305 10.1186/1556-276X-9-559 PMC4194061

[b33] González M. A. & Abascal J. L. F. The shear viscosity of rigid water models. *J*. *Chem*. *Phys*. 132, 096101 (2010). 20210414 10.1063/1.3330544

[b34] Harris K. R. & Woolf L. A. Temperature and volume dependence of the viscosity of water and heavy water at low temperatures. *J*. *Chem*. *Eng*. *Data* 49, 1064–1069 (2004).

