# Supplementary material for: Improved oil recovery in nanopores: NanoIOR
Source: Sci Rep. 2016 Jun 20;6:28128. doi: 10.1038/srep28128 (PMC4913300; doi:10.1038/srep28128)
Supplement: Supplementary Information [file srep28128-s1.pdf]

Supplementary material:
Improved oil recovery in nanopores: NanoIOR.

James Moraes de Almeida[1] and Caetano Rodrigues Miranda[1,2]

1: Centro de Ciências Naturais e Humanas, Universidade Federal do ABC, Santo André, SP, Brazil.

2: Instituto de Física, Universidade de São Paulo, São Paulo, SP, Brazil.

1. Structural data

Table S1: Number of atoms and system sizes for all the simulations.

Structure	Number of atoms	X size	Y size	Z size	Water Thickness	Oil Thickness
Silica SiOH Rich	46794	96.81	96.81	94.76	-	-
Silica SiOSi Rich	46320	96.81	96.81	94.37	-	-
Silica SiOH Rich + Water	100794	99.07	99.07	128.27	33.51	-
Silica SiOSi Rich + Water	100320	99.57	99.57	127.99	33.62	-
Silica SiOH Rich + Oil	116562	98.62	98.62	145.18	-	51.10
Silica SiOSi Rich + Oil	116088	98.64	98.64	146.51	-	52.78
Silica SiOH Rich + Water + Oil	170562	99.60	99.60	191.02	32.72	64.47
Silica SiOSi Rich + Water + Oil	170088	98.74	98.74	193.12	33.58	65.84

2. Interatomic Potential description

2.1 Silica:

The silica potential used was the Cruz-Chu, from reference: E. R. Cruz-Chu, *et al.* J. Phys. Chem. B, 110, 21497 (2006).

Naming:

We have adopted abbreviations for sake of simplicity, as follow in the next table.

Table S2: Abbreviations for the atomic names used.

Element	Name	Group Name
H_{SiOH}	SiOH Hydrogen	H_{Surf}
$H_{Si(OH)2}$	$Si(OH)_2$ Hydrogen	
O_{Bulk}	Bulk Oxygen	-
O_{SiOH}	SiOH Oxygen	O_{OH}
$O_{Si(OH)2}$	$Si(OH)_2$ Oxygen	
O_{SiOSi}	SiOSi Oxygen	-
Si_{Bulk}	Bulk Silicon	-

O_{SiOH}	SiOH Silicon	Si_{Surf}
$O_{Si(OH)2}$	$Si(OH)_2$ Silicon	

Non-bonded interactions:

The potential we used have the following functional form for the non-bonded and electrostatic interactions:

$$E_{LJ} = 4\epsilon\left[\left(\frac{\sigma}{r}\right)^{12} - \left(\frac{\sigma}{r}\right)^{6}\right], \qquad r < r_{C_{LJ}}$$

Where, ϵ and σ are the Lennard-Jones parameters, and $r_{C_{LJ}}$ is the cutoff for the explicit calculation of the interactions and r is the distance between the atoms. The used cutoff is 10 Å, with tail corrections.

$$E_{Coul} = \frac{Cq_iq_j}{r}, \qquad r < r_{C_{Coul}}$$

Where C is the Coulomb's constant, q_i and q_j are the charges of atoms i and j, r is the distance between the atoms and $r_{C_{Coul}}$ is the cutoff to explicitly calculate the interactions. The used cutoff was of 10 Å, the longer distances interactions were calculated in the reciprocal space with the pppm method.

In the following table the non-bonded interaction for the silica are shown:

Table S3: Lennard-Jones parameters and charges for the different atoms defined in the potential.

Element	ϵ	σ	Charge
H_{SiOH}	0.0460	0.400000	0.4320
$H_{Si(OH)2}$	0.0460	0.400000	0.4320
O_{Bulk}	0.1500	3.118145	-0.4500
O_{SiOH}	0.1521	3.153781	-0.6579
$O_{Si(OH)2}$	0.1521	3.153781	-0.6580
O_{SiOSi}	0.1500	3.118145	-0.4500
Si_{Bulk}	0.3000	3.826410	0.9000
O_{SiOH}	0.3000	3.826410	0.9010
$O_{Si(OH)2}$	0.3000	3.826410	0.9020

Bonded Interactions

Harmonic bonds:

The bonds were considered as harmonic interactions, with the following functional form:

$$E = K(r - r_0)^2$$

Where K is the spring constant and r_0 the equilibrium position. The parameters are as follow in the next table:

Table S4: Bond parameters of the silica potential.

Elements	K	r_0
O_{OH} – H_{Surf}	545.00	0.96
O_{OH} - Si_{Surf}	428.00	1.42

O_{Bulk} - Si_{Bulk}	885.10	1.61
O_{Bulk} - Si_{Surf}	885.10	1.61
O_{SiOSi} - Si_{Surf}	885.10	1.61
O_{SiOSi} - Si_{Bulk}	885.10	1.61
O_{OH} - O_{OH}	5.00	3.50

Harmonic Angles:

The angles were also considered as harmonic, the functional form is:

$$E = K(\theta - \theta_0)^2$$

Where K is the spring constant and θ_0 the equilibrium angle. The used parameters are as follows:

Table S5: Angle parameters for the silica potential.

Elements	K	Θ_0
Si_{Surf} - O_{OH} – H_{Surf}	57.50	106.00
Si_{Bulk} - O_{Bulk} - Si_{Bulk}	4.66	174.22
Si_{Surf} - O_{Bulk} - Si_{Bulk}	4.66	174.22
Si_{Surf} - O_{Bulk} – Si_{Surf}	4.66	174.22
O_{SiOSi} - Si_{Bulk} - O_{Bulk}	159.57	110.93
O_{SiOSi} - Si_{Bulk} – O_{SiOSi}	159.57	110.93
O_{SiOSi} - Si_{Surf} - O_{OH}	153.26	111.09

2.2. Water:

The used water potential was taken from Alejandre, J., *et al*. J. Chem. Phys. 130, 174505 (2009). All the parameters can be found in the reference.

2.3. Oil:

For Oil, the chosen potential was CHARMM, the reference with all the potential parameters can be found at: Brooks, B. R. et al. J. Comp. Chem. 30, 1545 (2009).

3. Velocity profile equilibration:

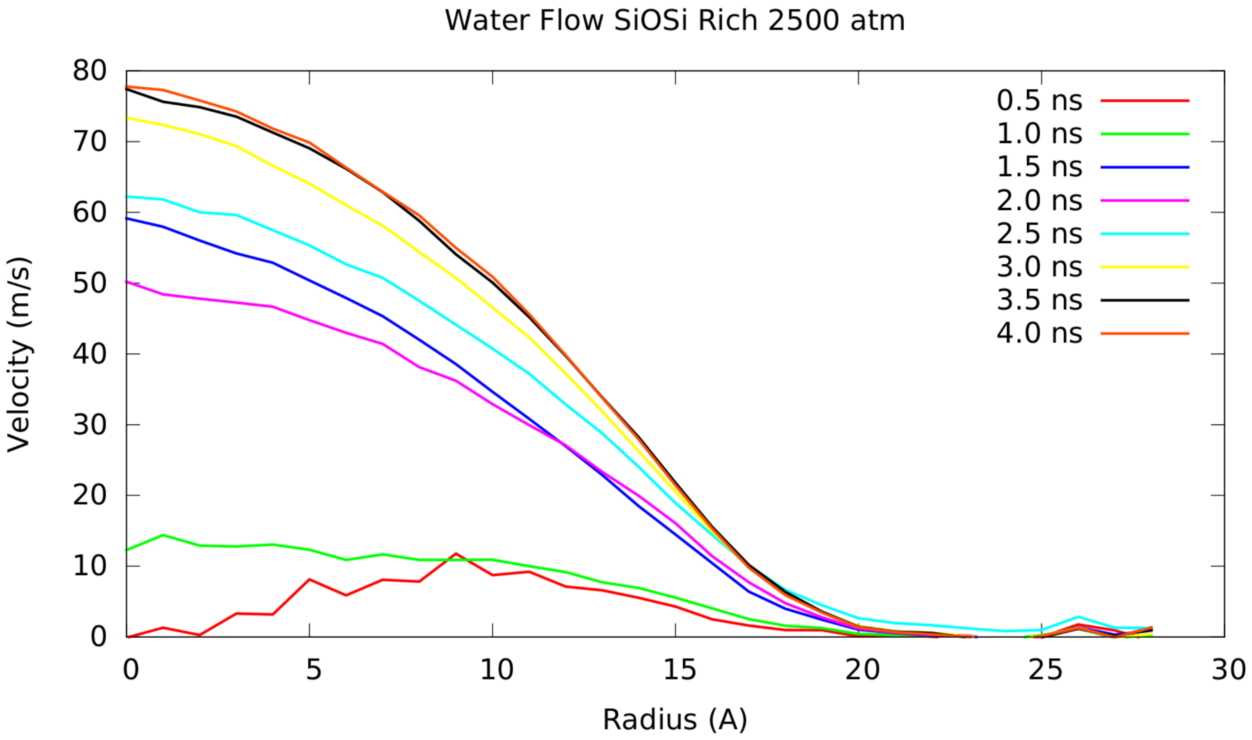

Figure S1: Radial velocity profile evolution along the equilibration of the flow.

4. Radial stress profile:

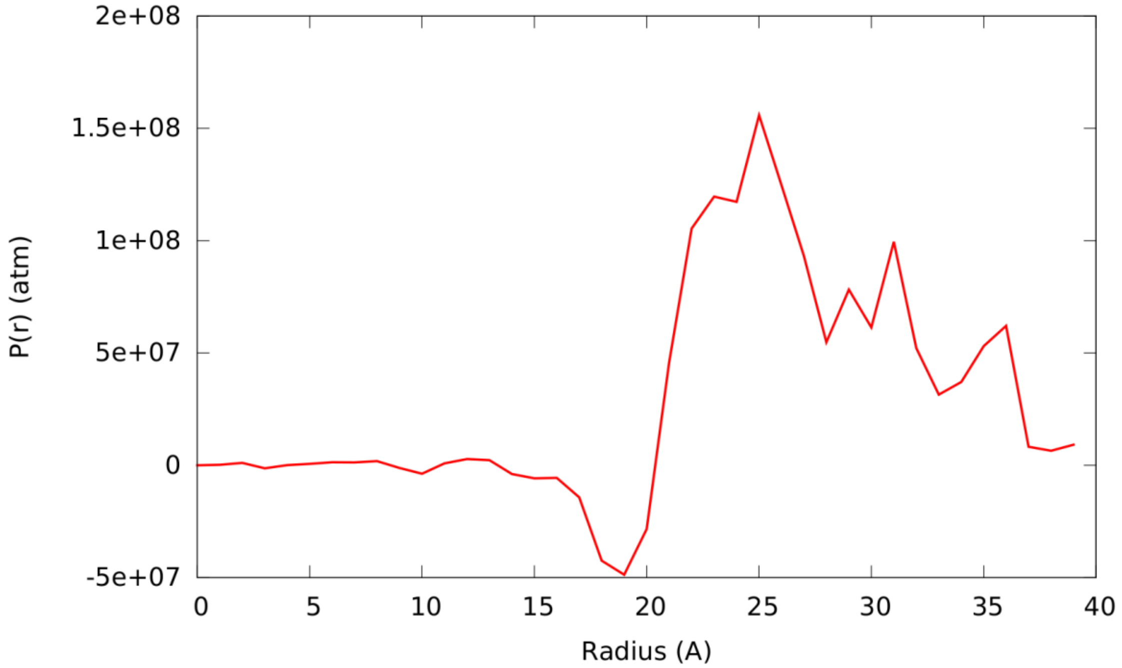

Figure S2: Radial stress profile from the center of the pore, crossing the interface and reaching the silica region.
